# Resilience and variability in soil nematode communities under nickel contamination: the mitigating role of olive trees

**DOI:** 10.1007/s10661-025-14435-2

**Published:** 2025-08-09

**Authors:** Aphrodite Theofilidou, Ioannis Zafeiriou, Panagiotis Kekelis, Vassilis Aschonitis, Dionisios Gasparatos, Nikolaos Monokrousos

**Affiliations:** 1 University Center of International Programmes of Studies, International Hellenic University, 57001 Thessaloniki, Greece; 2 Soil and Water Resources Institute, Hellenic Agricultural Organization—Dimitra, 57001 Thessaloniki, Greece; 3 Laboratory of Soil Science and Agricultural Chemistry, Agricultural University of Athens, 11855 Athens, Greece

**Keywords:** Ecological profile, Feeding groups, Functional groups, Soil pollution, Tolerant species

## Abstract

This study explores the effect of nickel contamination on the nematode community and assesses whether the presence of olive plants mitigates its impact. Soil samples were collected from both olive-cultivated and bare plots across a gradient of nickel concentrations (40, 70, and 120 ppm) in a Mediterranean agroecosystem. The results indicate that, even at high nickel concentrations (120 ppm), the presence of olive plants promoted the nematode community, while lower concentrations (40 ppm) favored the proliferation of bacterivorous nematodes (i.e., Panagrolaimus). The genus *Ditylenchus* exhibited resilience and dominance regardless of plant presence, while *Aphelenchus* also showed high abundance. Furthermore, plant presence maintained significantly higher biodiversity at 120 ppm compared to the intermediate concentration (70 ppm) in plant-absent conditions. In bare soils at 120 ppm Ni, nematode populations were entirely absent, while olive-cultivated soils still supported diverse communities, indicating a strong plant-mediated buffering effect. Diversity profiles and functional indices (maturity, enrichment, and structure index) revealed that olive trees not only buffered the negative effects of nickel but also promoted a more functionally diverse and stable nematode community, shifting dominance from fungivores to bacterivores at lower Ni concentrations. Multivariate analyses further demonstrated that both deterministic (Ni toxicity) and stochastic (plant-driven microhabitat heterogeneity) processes shaped community assembly, with olive trees enhancing resilience under stress. These findings highlight the importance of integrating plant-mediated remediation strategies in managing heavy metal-polluted soils and support the use of soil nematode communities as sensitive bioindicators for soil health assessment under environmental stress.

## Introduction

Heavy metal contamination in soils is a major environmental and agricultural issue with far-reaching consequences (Ma et al., 2024). Elevated concentrations of heavy metals in agricultural systems reduce crop yield, inhibit plant growth, and require immediate measures to mitigate soil toxicity (Li et al., 2014). Nickel (Ni) contamination, mostly due to anthropogenic activities such as mining and industrial operations, poses a significant threat to soil health and ecosystem stability (Gutiérrez et al., 2016). Long-term Ni accumulation alters soil quality by impacting beneficial soil organisms that interfere with nutrient cycling and degrading soil structure affecting soil fertility and resilience (Šalamún et al., 2012). Elevated Ni levels reduce both the population size and diversity of soil biota including microbes and nematodes which are vital for maintaining key ecosystem functions like organic matter decomposition and nutrient mineralization (Chauvin et al., 2020). These changes undermine plant-soil interactions and weaken the overall stability of soil food webs, leading to cascading ecological consequences (Gutiérrez et al., 2016). Declines in soil biota diversity and function reflect broader disruptions to the soil ecosystem since nickel toxicity compromises soil food web stability.

Understanding how nickel contamination impacts soil health and its associated biota is essential to the development of mitigation strategies for soil health restoration. Healthy soils rely on diverse microbial and faunal networks, which confer resistance and resilience to environmental perturbations (Morris & Blackwood, 2024). Nevertheless, disruptions of these networks mediated by heavy metal contamination include the dominance of opportunistic species and the loss of functional redundancy that lowers the ability of the soil to recover from stress (Ni). Thus, the study of shifts in these networks under nickel contamination could shed light on the dynamics of soil health and provide information on suitable remediation efforts.

Soil nematodes, being sentinel organisms, have proven to be good bioindicators of soil health (Martin & Sprunger, 2022), while their diminished population numbers and diversity in nickel-contaminated environments reveal the intensity of the problem (Chauvin et al., 2020). Nematodes are highly influential in the general functioning of terrestrial ecosystems, playing a crucial role in nutrient cycling and organic matter decomposition (Martin & Sprunger, 2022). Their sensitivity to changes in environmental conditions enables rapid responses to contamination and other disturbances in soil (Kekelis et al., 2022). Thus, their reduced presence in nickel-contaminated soils acts as an ecological signal of an imbalance, cascading into the impairment of plant health, nutrient cycling, and finally the resilience of the whole ecosystem (Harris et al., 2022). Indeed, nickel contamination affects not just nematodes but even the general soil biota through disruptive nets that maintain soil health in critical balance. Previous studies had documented that increasing concentrations of nickel result in a nematode population decline both as a direct result of their toxicity and from broader disruptions to ecosystem functioning (Chauvin et al., 2020; Dotaniya et al., 2020). These disruptions often result in reduced soil fertility, altered nutrient availability, and impaired plant growth, compounding the ecological impact of contamination. Few studies have assessed the effects of nickel contamination on the soil nematode communities of cultivated fields, making our understanding of the latter ecosystems rather poor (Douglas, 2012; Rudel et al., 2013).

The cultivation of plants in Ni-contaminated areas is challenging, as high concentrations of nickel in soil can severely affect plant growth, nutrient uptake, and lead to toxicity, ultimately resulting in low yield. However, olive trees (*Olea europea* L.) are very hardy trees that adapt to a wide variety of soils. 95% of the global surface of the olive trees is in the Mediterranean basin, where the pollution problem in olive orchards is often reported (Madejón et al., 2006; The International Olive Oil Council, n.d.). As mentioned, olive trees can withstand most of the environmental stressors, including heavy metal contamination, which makes it a hopeful candidate for the cultivation of contaminated areas with ecological and economic importance (Zaanouni et al., 2018). However, there is a research gap in addressing their role in mediating soil health under nickel stress, especially on their impact on soil microbial and faunal communities. Although the importance of soil health for sustainable agriculture is increasingly recognized, no study has yet evaluated the effects of nickel contamination on the structure and abundance of soil nematode communities in olive orchards. The study of such interactions may provide important information on how olive cultivation can affect soil health in contaminated landscapes and point out the potentiality of soil nematodes as bioindicators for monitoring and managing soil quality in these environments.

The composition and structure of nematode communities from Ni-contaminated soils are influenced by both deterministic and stochastic factors. Deterministic processes, such as soil pH, organic matter, and nutrient availability, may favor genera that have a greater chance to withstand nickel toxicity (Fiscus & Neher, 2002). In contrast, the more neutral or stochastic processes, such as random dispersal or ecological drift, could result in declines in nematode populations unexpectedly, contributing to variations in community dynamics, in such highly disturbed ecosystems (Lindo et al., 2023; Stamou & Papatheodorou, 2023). Current evidence suggests that deterministic processes often dominate in heavily contaminated environments due to strong selection pressures, though stochasticity may influence specific taxa or under certain conditions (Riddley et al., 2025). The presence of olive trees could mitigate these effects by altering soil properties and creating a more stable nematode community, thus promoting ecosystem resilience. Understanding these dynamics is essential for comprehending how Ni contamination affects soil ecosystems and identifying sustainable strategies to mitigate its impact.

This study aims to comprehensively investigate the impact of Ni contamination on soil nematode communities in agricultural ecosystems. To achieve this, samples were collected from both cultivated soils (planted with olive trees) and uncultivated soils (bare soil) across three Ni contamination gradients (120, 70, and 40 ppm). The primary goals were (i) to analyze how increasing Ni levels influence nematode abundance and community structure and to assess whether stochastic or deterministic processes govern nematode community assembly under varying contamination levels; (ii) to investigate the potential role of olive tree cultivation in mitigating the adverse effects of Ni contamination on nematode communities and whether the olive tree presence enhances community resilience by fostering functional diversity; (iii) to identify nematode genera or functional groups that exhibit resilience or susceptibility to Ni contamination.

## Materials and methods

### Study area

The study area extended from the National Park of Sounion at Agios Konstantinos to the French Wharf near the Port of Lavrio, covering approximately 5 km. This area encompassed both uncultivated and cultivated sites to evaluate and compare potentially toxic element (PTE) pollution, with a particular emphasis on its potential effects on soil health. Lavrio, situated approximately 60 km southeast of Athens, Attica, and Greece, spans an area of approximately 150 km^2^ and is characterized by a hilly to semi-mountainous terrain intersected by small streams. The region experiences a Mediterranean climate, with hot, dry summers. The mean annual temperature is 17.3 °C, ranging from a minimum of 4.2 °C in January to a maximum of 26.6 °C in July. The average annual precipitation is approximately 360 mm, with the highest rainfall recorded in December (85.2 mm) and the lowest in June (4.4 mm).

### Site selection and soil sampling protocol

**Map. 1:**
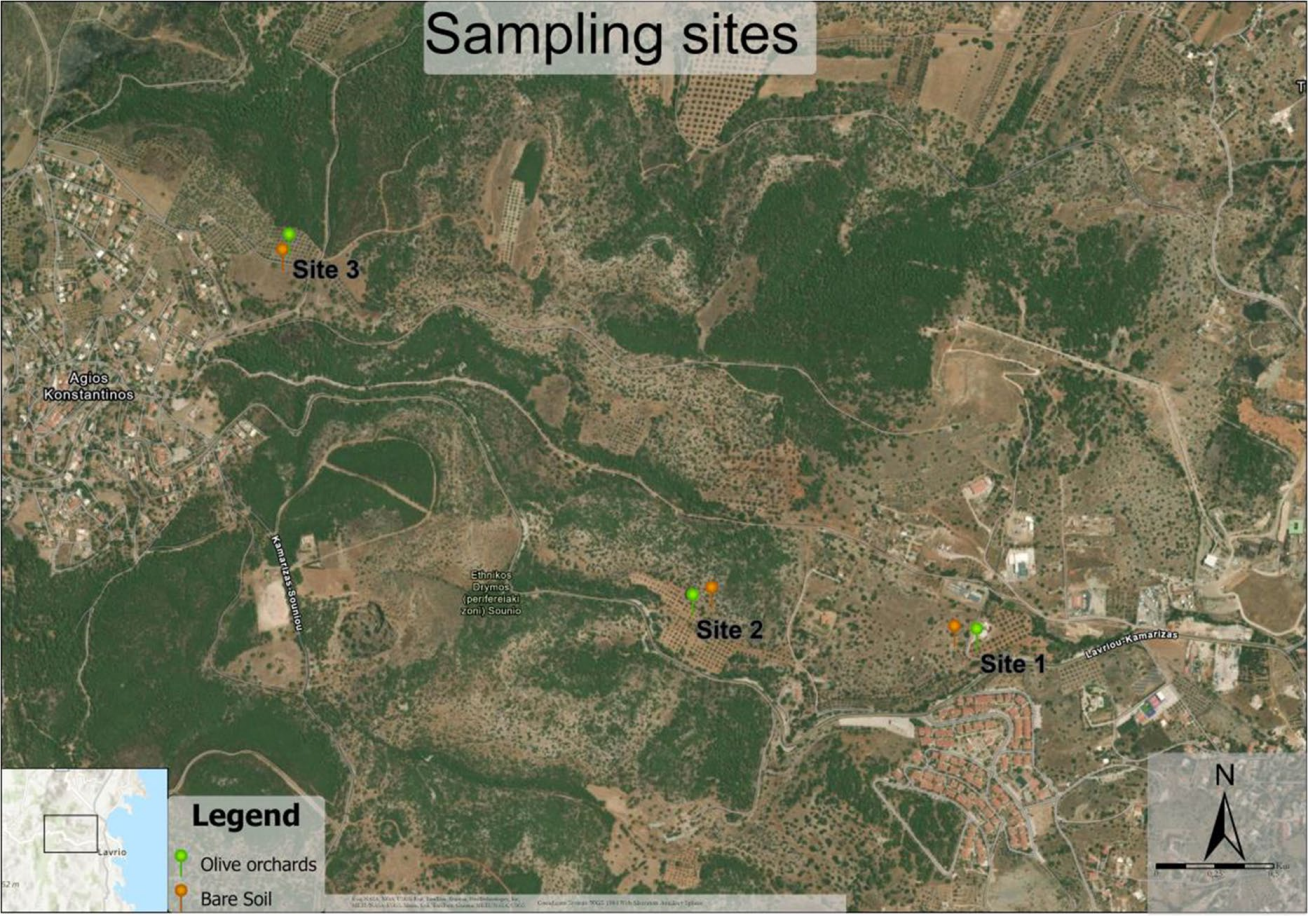
Soil sampling sites and land uses in the study area

For the present study, three sites were selected based on existing literature and previous studies conducted by the Soil Science and Agricultural Chemistry Laboratory of the Agricultural University of Athens (Kalyvas et al., 2018; Zafeiriou et al., 2019). The selected sites were chosen to represent a gradient of soil nickel (Ni) concentrations, while maintaining similar levels of other soil physio-chemical properties and PTEs concentration. Prior to the main sampling campaign, a preliminary exploratory sampling was conducted to confirm the suitability and representativeness of the selected sites for the intended comparative analysis. Following this confirmation, in October 2022, comprehensive soil sampling was carried out across the three validated sites. At each site, two paired land-use types were identified in close proximity: olive orchards and bare soil. The bare soil plots were defined as uncultivated areas completely free of vegetation cover, with care taken to exclude patches of natural or residual plant growth. In contrast, soil sampling in the olive orchards was conducted within the canopy zone, close to the trunk, with each sampling plot including approximately 3–4 olive trees. The geographic coordinates of the selected sites are as follows: site 1: N 37.7211°, E 24.0422°, site 2: N 37.723045°, E 24.03259° site 3: N 37.723284°, E 24.01831° with an overview of the site locations and land uses being presented in Map 1. At each site, a minimum homogeneous area of 0.25 ha was identified for each land-use type. Within these areas, four independent sampling plots of 100 m^2^ each were established per land use. The plots were spaced at least 20 m apart to ensure spatial representativeness and to capture intra-site soil heterogeneity. From each plot, five individual soil cores were collected to a depth of 20 cm using a cylindrical soil sampler with a diameter of 5 cm. A random zigzag sampling pattern was followed. Prior to sampling, all surface debris, such as weeds, dry leaves, plant residues, and gravel, was removed. The five cores from each plot were thoroughly homogenized in the field to generate a composite sample of approximately 1 kg. This protocol was consistently applied across both land-use types at all three sites, yielding a total of 24 composite samples (3 sites × 2 land uses × 4 replicates). All samples were placed in airtight plastic bags immediately after homogenization and stored at 4 °C until further laboratory analysis.Map 1Soil sampling sites and land uses in the study areaMap 1Soil sampling sites and land uses in the study areaMap 1Soil sampling sites and land uses in the study area

### Soil physicochemical parameters

The majority of soil samples were characterized as sandy loam (SL), indicating a moderately coarse texture. The study area was segregated based on Ni–concentration (Table 1) and nematode diversity evaluation was performed according to that.

**Table 1:** Ni–concentration in the soil in different sites of the study area

Soil site	Ni (ppm)	Olive orchards
1	120	Presence
		Absence
2	70	Presence
		Absence
3	40	Presence
		Absence

To determine the physicochemical soil properties, the samples were air-dried at room temperature, crushed, sieved through a 2-mm sieve and analyzed for the following parameters: pH was measured in a suspension with a 1:1 (w/v) ratio of soil to distilled water with a pH meter; electrical conductivity (EC) was determined in the soil paste extract (Page, 1982) and soil organic matter (SOM) by dichromate oxidation according to Walkley–Black’s procedure (Nelson & Sommers, 1982). Particle size distribution was determined by the hydrometer method (Bouyoucos, 1951), while equivalent calcium carbonate percentage was calculated by measuring the evolved CO_2_ following HCl dissolution (AFNOR, 1995). Cation exchange capacity (CEC) was determined by the ammonium acetate method (Rhoades, 1982) and exchangeable K^+^ was extracted using CH_3_COONH_4_ 1 M, pH = 7 (Page, 1982). The total contents of the PTEs extracted with aqua regia were determined in an atomic absorption spectrophotometer (Varian—spectra A300 system) (Gasparatos & Haidouti, 2001), while bioavailable fraction with single-step DTPA extraction, as described by Lindsay and Norvell (1978).

### Nematode extraction, identification, and analysis

Nematodes were extracted from a volume of 150 ml of soil for each sample, following an initial manual disintegration of soil aggregates to minimize structural disruption. The extraction was performed using Cobb’s sieving and decanting method, as modified by s’Jacob and Van Bezooijen (1984). After enumeration, nematodes were preserved in 4% formaldehyde. A random subset of 100 nematodes from each sample was then identified to the genus level using Bongers’ taxonomic key (Bongers, 1994). The identified genera were classified into trophic groups based on the criteria established by Yeates et al. (1993) and further categorized with c–p values (colonization-persistence) (Bongers, 1990; Bongers & Bongers, 1998). Predators and omnivores were combined into a single group because both were present in very low abundances in our samples, making separate statistical analysis unreliable. This approach is supported by their similar ecological roles as higher-level consumers within the soil food web (Yeates et al., 1993), and is commonly adopted in studies with low numbers of these groups (Mo et al., 2024; Wang et al., 2022, 2024). Furthermore, the term “herbivores” includes both plant parasitic nematodes and non-parasitic plant feeders (i.e., all phytotrophs), as this grouping provided a more robust dataset for analysis and better reflected the functional impact on plant communities. To assess nematode functional indices, the maturity index (MI) was calculated for free-living nematodes, reflecting the balance of tolerant versus sensitive species and indicating the successional stage of the community (Bongers, 1990). The plant parasitic index (PPI) was determined exclusively from the abundances of plant parasitic nematodes, reflecting the relative dominance of these taxa and their potential impact on plant health (Bongers, 1990). Additionally, the enrichment index (EI) and structure index (SI) were calculated following Ferris et al. (2001) and are considered together as indicators of soil food web enrichment and structuring. EI reflects the response of opportunistic nematodes to resource enrichment, while SI indicates the degree of trophic complexity and the presence of higher c–p value omnivores and predators, thus reflecting soil food web stability. The channel index (CI) was calculated separately, as it distinguishes between bacterial and fungal decomposition channels, indicating the dominant pathway of organic matter breakdown in the soil (Ferris et al., 2001). Finally, the NINJA (Nematode-Indicator Joint Analysis) online platform was employed to calculate metabolic footprints (bacterivore footprint: BF and fungivore footprint: FF) and generate a graphical representation of the soil food web (Sieriebriennikov et al., 2014).

### Statistical analysis

Two-way analysis of variance (ANOVA) was conducted to evaluate the effects of treatment (presence or absence of olive trees), Ni concentration, and their interaction on soil nematode populations and nematode community indices. Prior to ANOVA, the normality of residuals was assessed using the Shapiro–Wilk test, and homogeneity of variances was tested using Levene’s test. Where necessary, data were log-transformed to meet these assumptions, a standard approach in soil ecology. Mean comparisons were performed using Fisher’s Least Significant Difference (LSD) test, with significance set at *p* < 0.05. Non-metric multidimensional scaling (NMDS) was implemented in RStudio to assess the impact of Ni concentration and olive tree presence on nematode community structure. The relative abundances of nematode genera were used to position communities within a multidimensional space based on the Bray–Curtis distance coefficient. Additionally, a Similarity Percentage (SIMPER) analysis was performed to determine the contributions of different genera to community dissimilarity (Kruskal, 1964). Redundancy Analysis (RDA) was also conducted in RStudio to explore correlations between nematode genera and soil properties. All statistical analyses were carried out using the statistical software R (4.2.2) and R – Studio (12.0.0) with “agricolae,” “ggplot2,” “readxl,” “vegan,” and “dplyr” packages for statistical analysis and visualization (Ushey et al., 2020).

To assess nematode community diversity, we applied the diversity ordering method proposed by Patil and Taillie (1979), utilizing Rényi’s diversity index (Rényi, 1961). This parametric index, defined by the order parameter *α*, allows for varying sensitivity to rare and dominant species within a community (Ricotta, 2000). The method generates a diversity profile widely used in ecological studies. Specifically, when *α* = 0, the index corresponds to the logarithm of species richness; at α = 1, it is equivalent to Shannon’s index, while at *α* = 2, it corresponds to Simpson’s index. As *α* approaches infinity, the index becomes increasingly influenced by the most abundant species. Differences in diversity at lower *α* values reflect variations in species richness, whereas those observed at higher α values indicate differences in species dominance. When diversity profiles of two communities intersect, it suggests that their ranking may vary depending on the diversity measure used. All calculations were performed using PAST 4.03 software.

## Results

**Fig. 1:**
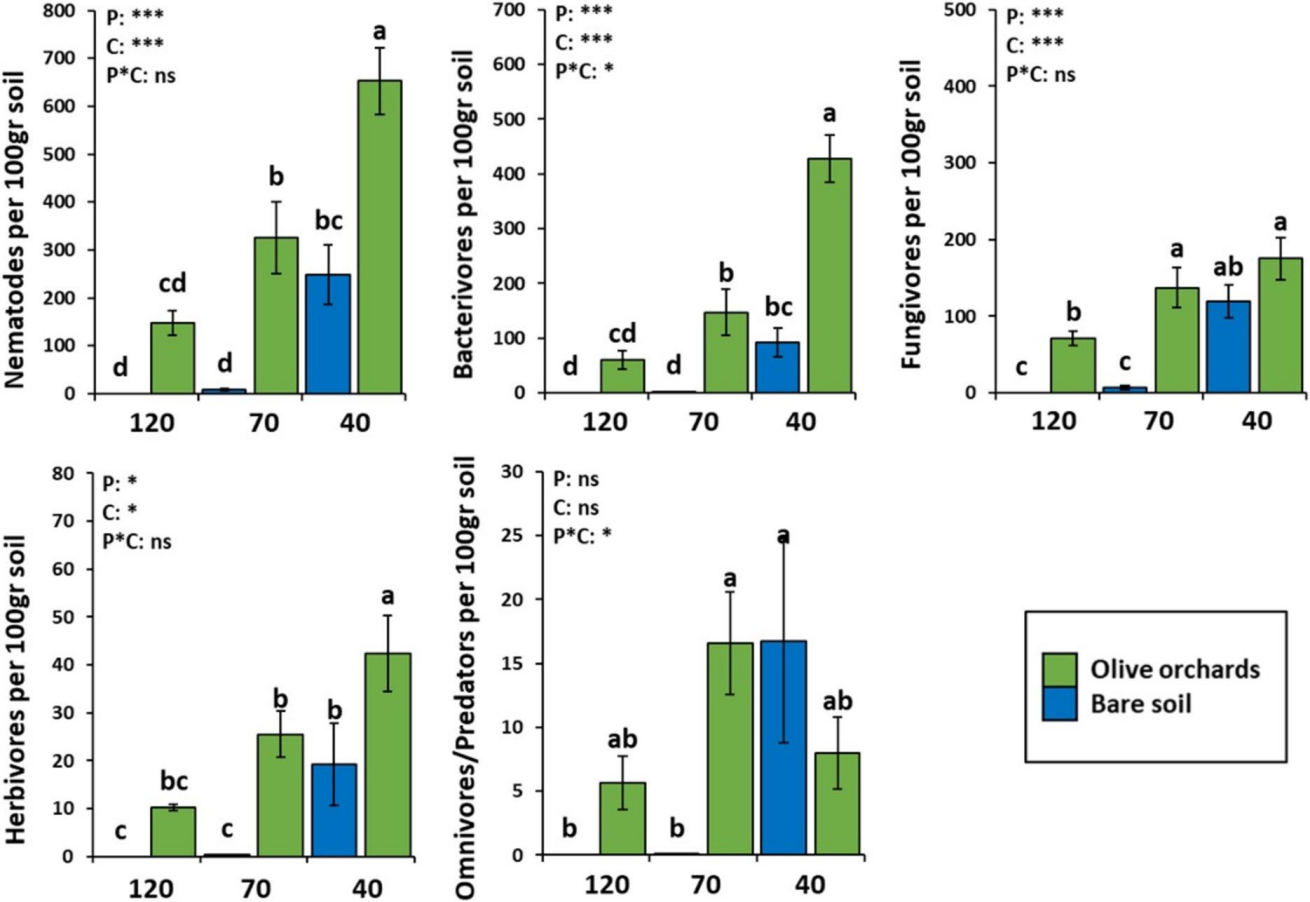
Mean abundance values (± standard error) of nematode trophic groups (total nematode abundance, bacterivores, fungivores, herbivores and omnivores/predators) under different concentrations and ANOVA results regarding the presence or absence of plant (P: plant) and nickel (Ni) concentration in the soil (C: concentration). Different letters (a, b, c, d) indicate significant differences between treatments based on Fisher’s LSD post hoc test (*: *p* < 0.05, ***: *p* < 0.001, ns: not significant, for all cases *n* = 4

The abundance of each nematode trophic group is presented in Fig. 1. At nickel concentrations exceeding 120 ppm in uncultivated bare soil, no nematodes were documented. At 70 ppm, the total nematode population was low (9.1 ± 2.6 nematodes per 100 g soil), while the lowest concentration (40 ppm) exhibited a notably higher abundance (247.8 ± 61.8 nematodes per 100 g soil). A similar trend was observed in olive orchards; however, the abundances were markedly higher in the presence of olive trees. The reduction in nematode numbers with increasing nickel concentration was significant in uncultivated soils, while the presence of plants substantially mitigated this effect. The most pronounced increase in nematode abundance occurred at the lowest nickel concentration, primarily driven by a corresponding rise in bacterivores. This abundance pattern was also evident, albeit less pronounced, in herbivores, while fungivores showed similar results at 70 and 40 ppm in olive orchards. Notably, statistically significant differences in omnivores/predators were only observed in uncultivated soil at high nickel concentrations, where their values were nearly zero.

**Fig. 2:**
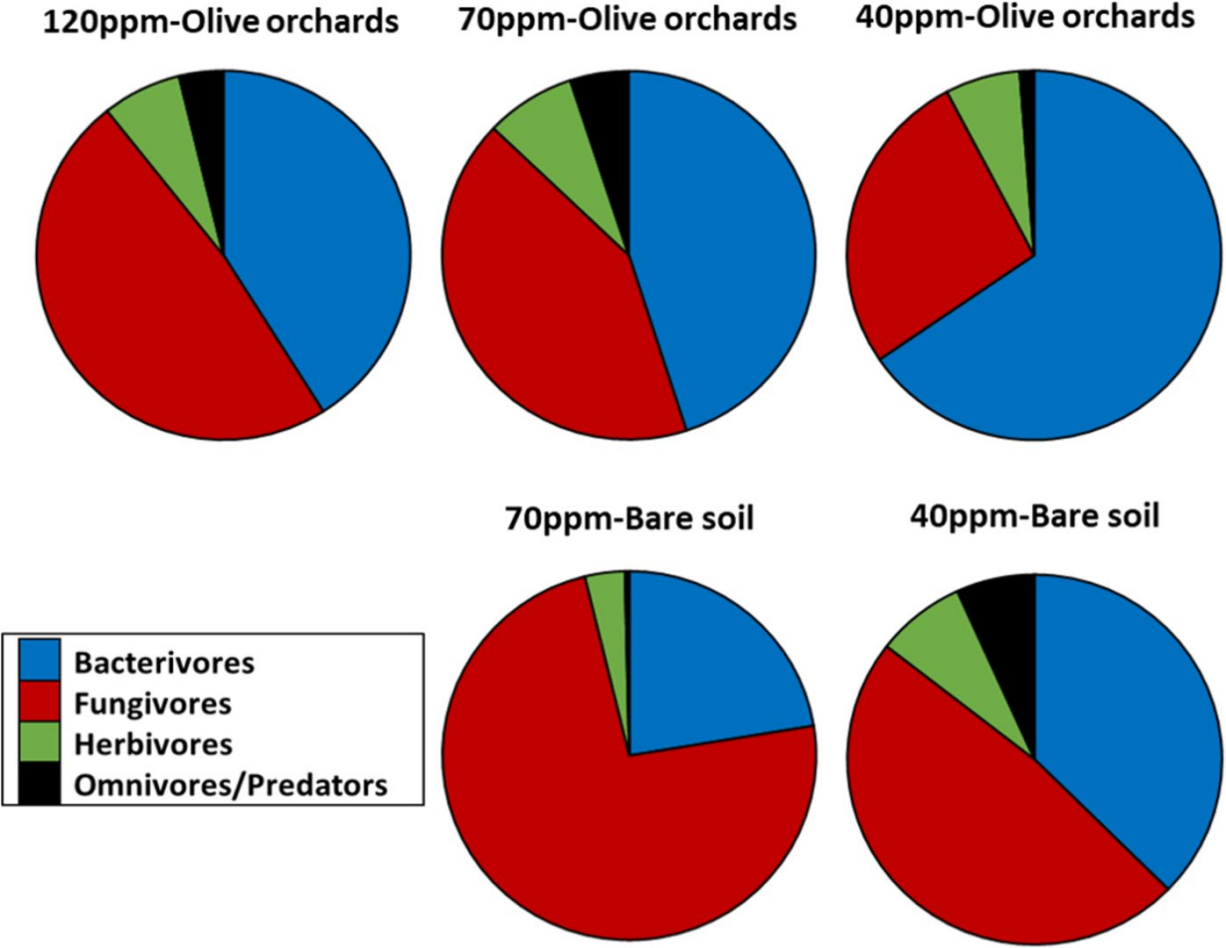
Percentage contribution of nematode trophic groups at different Ni concentrations in uncultivated-bare soils and olive orchards. For all cases *n* = 4

The composition of nematode populations exhibited structural similarity at high and medium nickel concentrations (> 120 and 70 ppm) in the presence of plants, while at the lowest concentration (40 ppm), an increase in bacterivores was observed in Fig. 2. In uncultivated soil with medium nickel concentrations (70 ppm), fungivores were dominant, and omnivores/predators were notably absent. At low concentrations (40 ppm), omnivores/predators appeared, and a slight increase in the abundance of bacterivores was observed. Notably, at very high concentrations in the absence of plants, nematodes were completely absent.

**Fig. 3:**
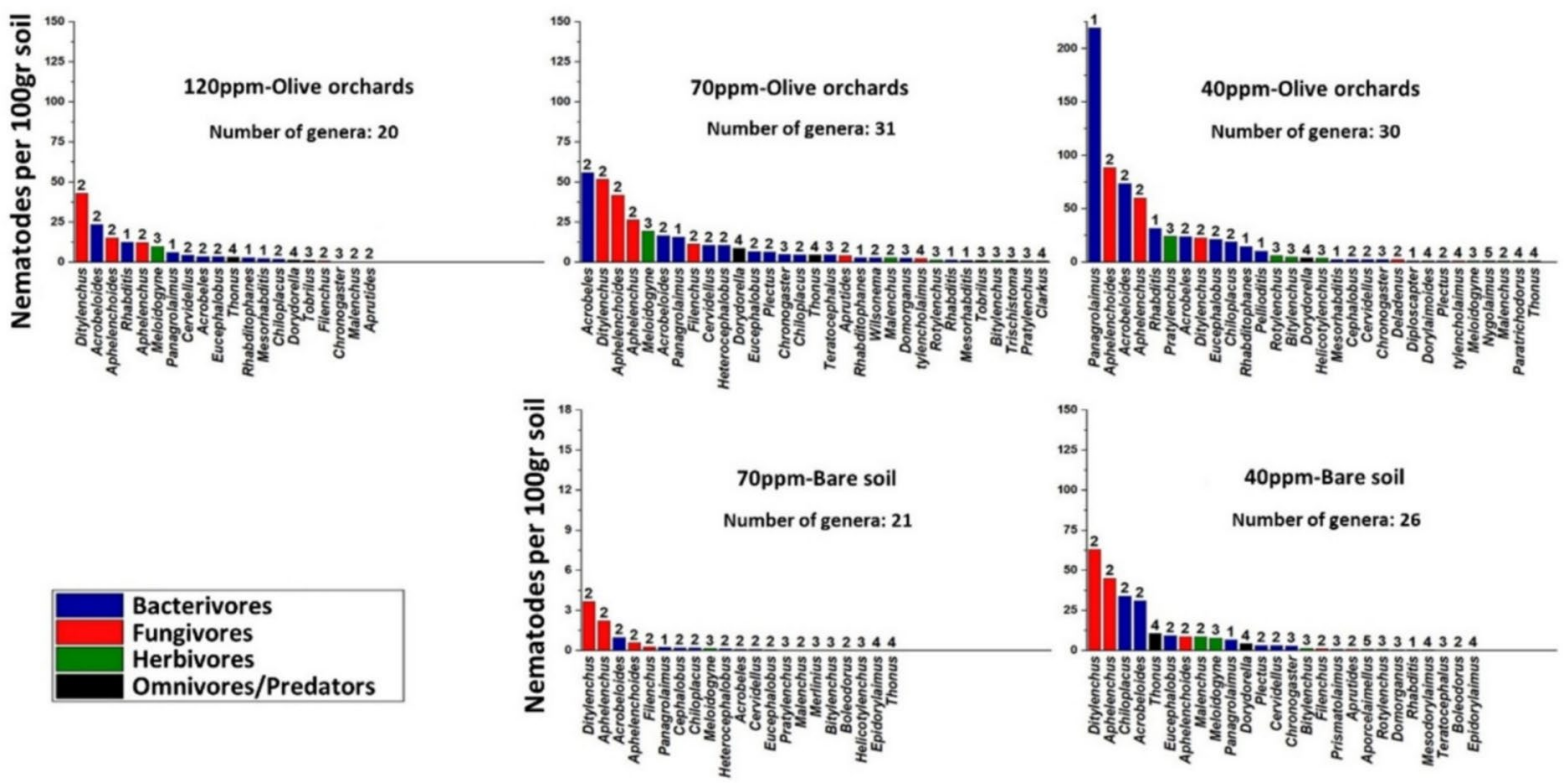
Rank abundance of nematode genera in different cases. The numbers above the bars reveal the survival strategy of each genus on the cp scale, where “1” refers to short-lived and rapidly-growing genera, and “5” refers to longer-lived and large-body genera

The escalation of nickel (Ni) concentrations, as presented in Fig. 3, correlated with an increased prevalence of the fungivorous cp-2 genus *Ditylenchus*, a trend consistently observed across all treatments involving uncultivated soil. Specifically, while *Ditylenchus* achieved high abundance at medium and low concentrations (70 ppm and 40 ppm) in bare soil, it was not distinctly dominant, as the fungivorous cp-2 genus *Aphelenchus* also maintained a notable presence, resulting in a more evenly distributed bio-community. In olive orchards, a similar distribution pattern emerged at the highest Ni concentration (120 ppm), with *Ditylenchus* maintaining dominance. However, as Ni concentrations decreased to 70 ppm, the bio-community composition in olive orchards became more even, with the cp-2 bacterivore *Acrobeles*, cp-2 fungivore *Ditylenchus*, and cp-2 fungivore *Aphelenchoides* reaching comparable abundances. This even distribution shifted notably at the lowest Ni concentration (40 ppm), where the bacterivorous cp-1 enrichment opportunist genus *Panagrolaimus* became over-dominant.

**Fig. 4:**
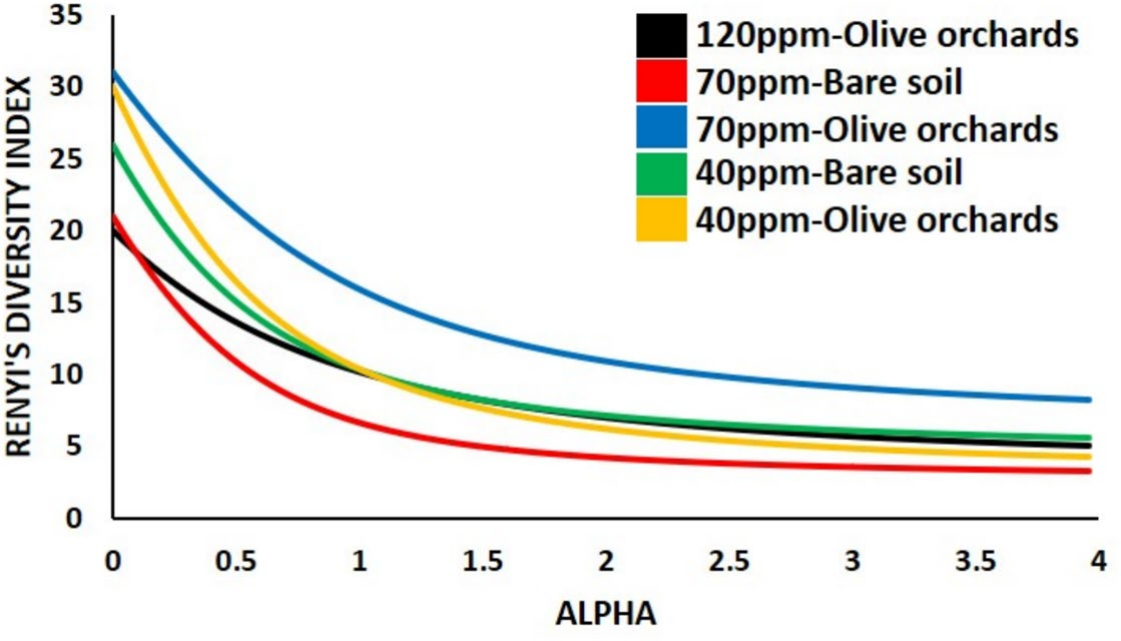
Diversity profiles of nematode communities in the five Sects. (120-, 70-, 40-ppm olive orchards and 70- and 40-ppm bare soil). For *a* = 0, 1 and 2, the index is equal to the genus richness, Shannon index and Simpson index, respectively, while a > 2 presents the evenness of the bio-community

The biodiversity assessment revealed that the majority of genera, along with higher Shannon index values (Fig. 4), were recorded in soil with a nickel concentration of 70 mg kg-1 in the presence of olive trees. In contrast, the lowest diversity indices were observed in uncultivated, bare soils. The escalation of heavy metal concentrations in soil has been shown to significantly reduce both the abundance of nematodes and the diversity of genera. Notably, in olive orchards with a 40-ppm nickel concentration, a low evenness was observed for alpha > 2, despite the highest number of genera (alpha = 0). This was attributed to the over-dominance of the cp-1 enrichment opportunist *Panagrolaimus*, which led to an unstable nematode community structure.

**Fig. 5:**
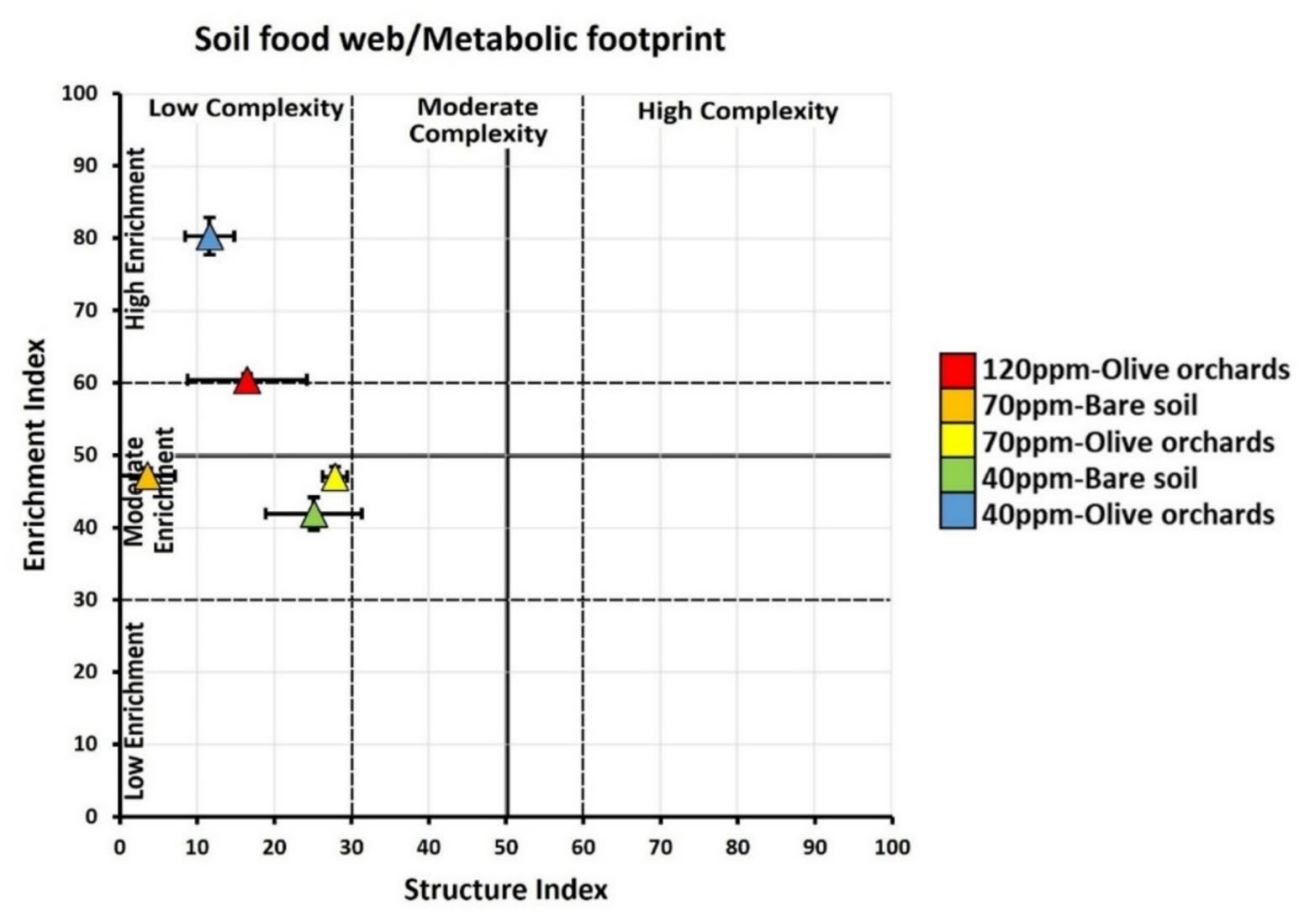
Food web analysis according to the ordination of the samples based on the EI and SI values of different Ni concentrations (> 120, 70, and 40) and in the presence (olive orchards) or absence (bare soil) of olive trees. The triangles point to the mean value of the ratio and the bars show the respective standard error. For all cases *n* = 4

The examination of the food web, based on the enrichment index (EI) and structure index (SI) values, in treatments involving nickel and plant presence is presented in Fig. 5. Variations in nickel concentration within the soil cultivated with olive orchards impart distinct characteristics. Elevated nickel levels contributed to soil disturbance, while lower concentrations created an environment more conducive to growth. Additionally, this soil exhibited nitrogen enrichment, a reduced C:N ratio, and a discernible bacterial footprint. These nuanced impacts highlight the intricate interplay between nickel levels and soil quality in olive orchard cultivation. At 70 ppm Ni, the soil experienced degradation and depletion, fostering conditions favorable for specific growth patterns. The soil presented an elevated C:N ratio, indicating altered composition, as well as a discernible fungal footprint.

The nematode indices are presented in Table 2. The MI index exhibited its maximum values at 40 ppm in bare soil (2.1 ± 0.04) and 70 ppm in olive orchard soil (1.6 ± 0.05). High pollution levels induced a noticeable decline in the MI index. The PPI and EI reached their minimum at 40 ppm in bare soil (2.4 ± 0.25), with higher values observed in the other treatments. EI peaked at 40 ppm in olive orchards (80.3 ± 2.59). The CI and SI indices reached their minimum values at the low concentration (40 ppm) in olive orchards (13.7 ± 2.3 and 11.6 ± 3.15, respectively) while the presence of plants seemed to increase their values at higher Ni concentrations (44.2 ± 3.09 and 16.5 ± 7.74, respectively). Both the fungivore and bacterivore footprints exhibited the lowest values at 70 ppm in bare soil (1 ± 0.32 and 0.6 ± 0.19, respectively) and the highest at 40 ppm in olive orchards (17.7 ± 3.56 and 189.2 ± 22.03, respectively). In all cases, high contamination in the absence of plants showed no data, so it is not included.

**Table 2:** Mean values (± standard error) of the maturity index (MI), enrichment index (EI), channel index (CI), plant parasitic index (PPI), and structure index (SI) under the different contamination levels (> 120, 70, and 40 ppm Ni), in both bare soil and olive orchards’ soil. The final three rows indicate significance for each factor: Plant presence of abscense (Pl), contamination level (Cont), and their interaction (Pl*Cont), as revealed by ANOVA and Fisher’s LSD post hoc comparisons (*p < 0.05; **p < 0,01; ***p < 0.001; ns, not significant, for all cases *n* = 4). Within each column, values sharing the same letter are not significantly different

Cont	Plant	MI	PPI	CI	EI	SI	FF	BF
120 ppm	Bare soil	0 ± 0^**e**^	0 ± 0^**c**^	0 ± 0^**e**^	0 ± 0^**e**^	0 ± 0^**d**^	0 ± 0^**b**^	0 ± 0^**c**^
	Orchards	1.9 ± 0.05^**c**^	3 ± 0.02^**a**^	44.2 ± 3.09^**c**^	60.4 ± 0.86^**b**^	16.5 ± 7.74^**abc**^	10.4 ± 1.38^**a**^	39.4 ± 12.39^**b**^
70 ppm	Bare soil	2 ± 0.01^**bc**^	2.9 ± 0.1^**a**^	85.9 ± 3.32^**a**^	47.1 ± 0.98^**c**^	3.6 ± 3.56^**cd**^	1 ± 0.32^**b**^	0.6 ± 0.19^**c**^
	Orchards	2.1 ± 0^**ab**^	2.9 ± 0.05^**a**^	62.5 ± 3.91^**b**^	47 ± 1.34^**c**^	27.8 ± 1.57^**a**^	16.4 ± 3.78^**a**^	35.4 ± 8.7^**b**^
40 ppm	Bare soil	2.1 ± 0.04^**a**^	2.4 ± 0.25^**b**^	84.1 ± 8.12^**a**^	41.9 ± 2.24^**d**^	25.1 ± 6.2^**ab**^	16.9 ± 2.93^**a**^	24.3 ± 5.74^**bc**^
	Orchards	1.6 ± 0.05^**d**^	3 ± 0.03^**a**^	13.7 ± 2.3^**d**^	80.3 ± 2.59^**a**^	11.6 ± 3.15^**bcd**^	17.7 ± 3.56^**a**^	189.2 ± 22.03^**a**^
	**Plant**	***	***	***	***	*	*	***
	**Cont**	***	***	***	***	**ns**	*	***
	Pl*Cont	***	***	***	***	**	*	***

**Fig. 6:**
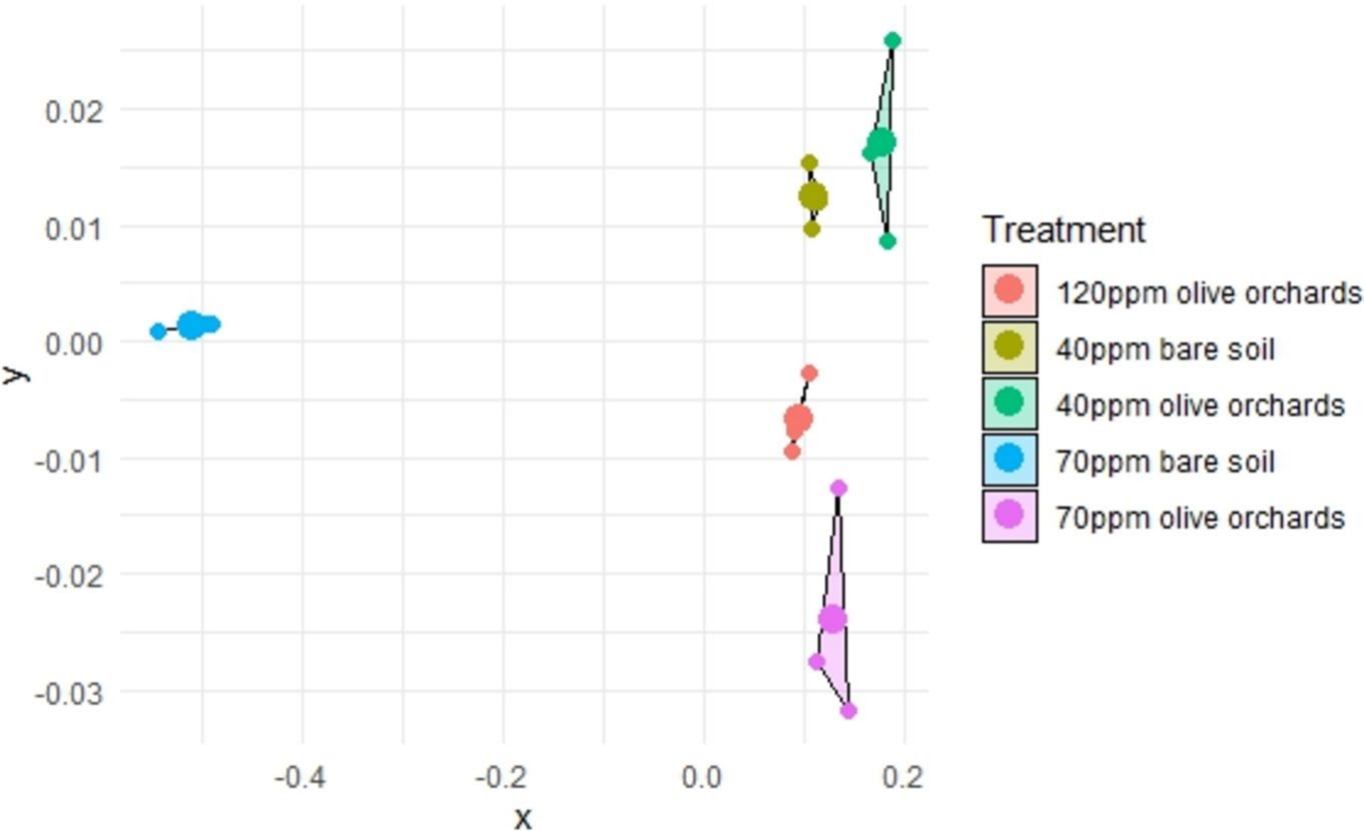
A non-metric multidimensional scaling (NMDS) plot shows changes in nematode communities’ genera composition due to contamination and plant presence. The shorter the distance linking two samples, the higher the similarity between them. Different colors stand for different treatments (*n* = 4)

In the NMDS graph (Fig. 6), a greater distance between circles indicates a higher degree of community differentiation. The ordination of 70 ppm contamination in bare soil stands out as the most distinct, with clear differentiation from the other treatments. This separation can be attributed to the co-dominance of two cp-2 fungivorous genera, *Ditylenchus* and *Aphelenchus*, with few other community members present. In contrast, 40 ppm contamination in olive orchards and 120 ppm and 70 ppm in olive orchards formed a cluster, showing no significant differences in the nematode community. The differentiation in olive orchards, with 40 ppm contamination, was driven by the over-dominance of the cp-1 bacterivore *Panagrolaimus*.

The Simper test (Table 3) revealed that *Ditylenchus* was the primary contributor to the difference between highly contaminated cultivated soil (120 ppm Ni) and the other treatments. On the other hand, *Panagrolaimus* was the primary contributor to the differentiation at the lowest contamination (40 ppm Ni) in olive orchards. 

**Table 3:**
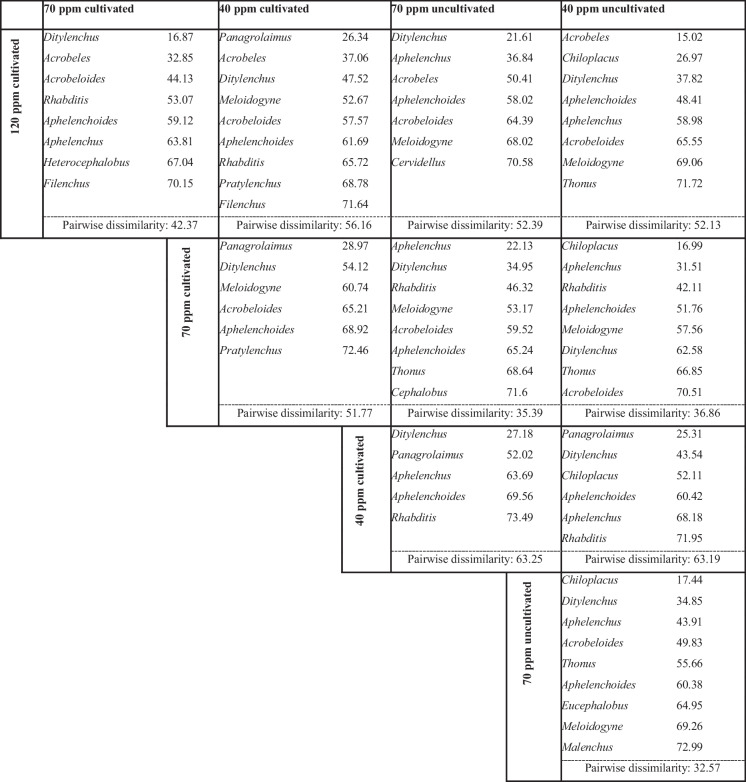
Dissimilarity percentages between treatments and results of SIMPER test (similarity percentage) based on nematode genera abundances. Nematode genera accounting for > 70% of overall dissimilarity are ranked in order of importance of their contribution (n = 4)

**Fig. 7:**
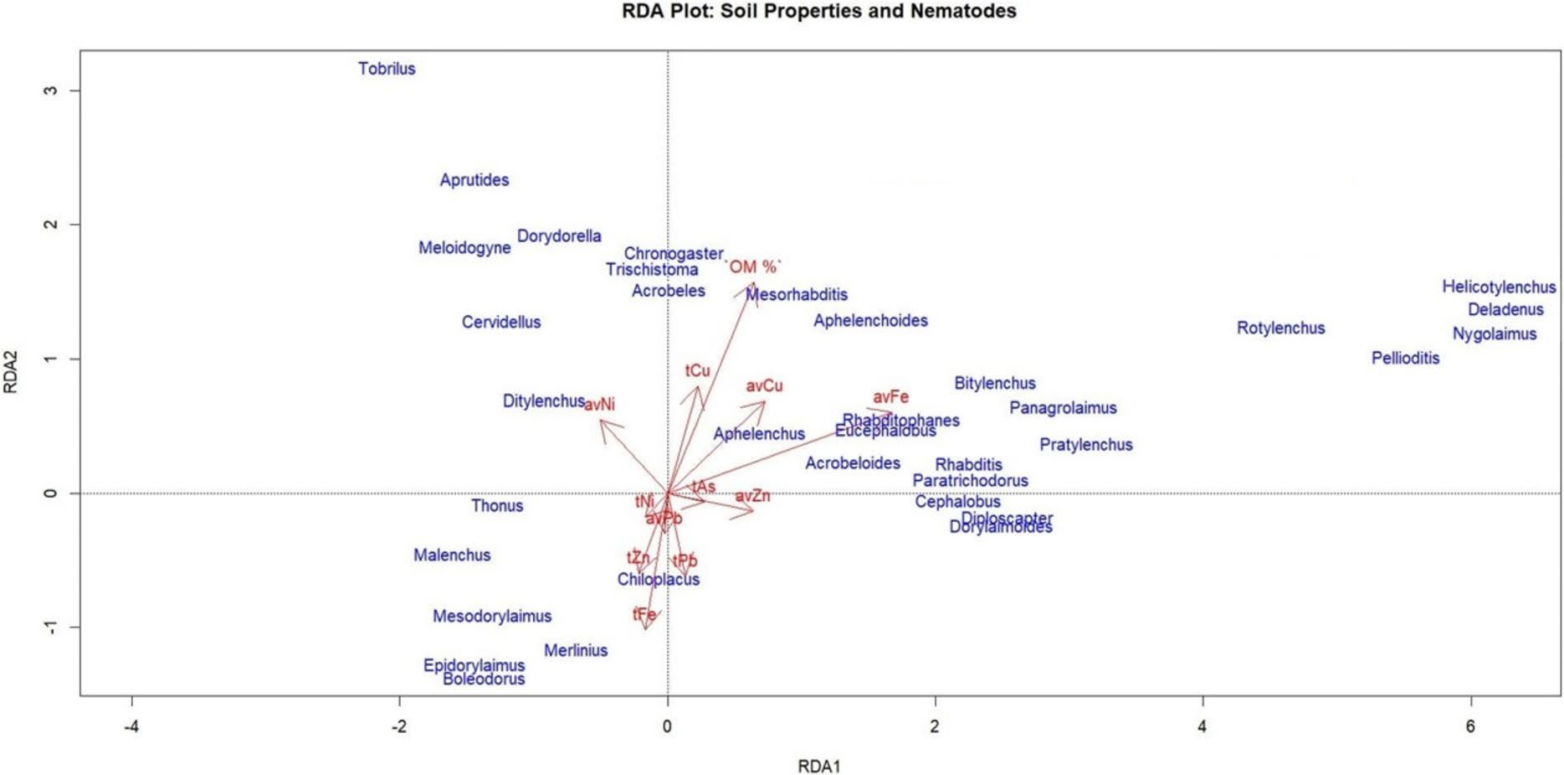
Constrained redundancy analysis (RDA) displaying contributions of soil properties (red-colored lines and letters) and nematode genera (blue-colored letters) (*n* = 4). Soil properties include both the total and the available concentration of each element (t: total concentration; av: available concentration)

The RDA plot highlights the significant influence of soil properties and heavy metals on nematode community composition (Fig. 7). The RDA model explains 32.7% of the total variance along the first two axes. The cp-2 fungivore genus *Ditylenchus* was clustered near the available Ni vectors an indication of resilience to Ni contamination. On the contrary, most bacterivores were ordinated away from heavy metal vectors, indicating effects of Ni toxicity, but are strongly associated with organic matter content (OM%). More specifically, organic matter explained 12.02% of the variance on RDA1 and 78.84% on RDA2; available iron explained 80.63% of the variance on RDA1 and 11.56% on RDA2; and available nickel explained 7.34% on RDA1 and 9.57% on RDA2.

## Discussion

Our study provides significant insights into the complex interactions between the dynamics of soil nematode communities, plant-mediated impacts, and nickel contamination. These findings provide important implications for sustainable soil management in Ni-contaminated environments by demonstrating the combined influence of deterministic and stochastic processes on soil ecosystems.

### Deterministic and stochastic effects of nickel contamination and olive trees on nematode communities

In both cultivated and bare soil systems with lower Ni concentrations, abundance and genus richness of nematodes increased significantly. This was a clear indication of a deterministic effect of nickel toxicity, in which increasing contamination levels suppress nematode populations. Interestingly, in uncultivated soils above 120 ppm Ni, the complete absence of nematodes further complies with an Ni-dose-dependent effect (Tóth et al., 2016).

Moreover, the responses of trophic group categories also reflected deterministic processes shaping community structure at high Ni concentrations in both systems. In bare soil samples, these were evident across all Ni concentration levels; fungivorous nematodes dominated the community structure, as Ni seemed to impact mostly the bacterial feeders. These findings underscored the differing ecological tolerances of these groups. *Ditylenchus* exhibited a remarkable capacity to thrive under heavy metal stress, likely due to physiological adaptations that confer resistance to nickel toxicity, enabling its survival and dominance (Jiang et al., 2023). Even though there is limited evidence to suggest that bacterivores experience a direct sensitivity to Ni toxicity, the fact that the bacterial feeders were found at low abundances is probably due to the indirect impact of toxic effects on bacterial populations, which serve as their primary food source (Zeng et al., 2020). High Ni levels can suppress bacterial activity and biomass, disrupting the base of the soil food web. This reduced availability of resources indirectly affects bacterivores, leading to a decline in their abundance.

Despite the clear deterministic trends in the bare soil samples, the presence of olive trees introduced additional variability to nematode community responses. Firstly, nematode populations were significantly higher compared to those recorded in uncultivated bare soils at all contamination levels. Even at high Ni concentrations (120 ppm), olive trees were able to support small but viable nematode populations, which were absent in bare soils. This finding highlights the role of olive trees in mitigating the negative effects of increased Ni contamination contributing significantly to the resilience of the nematode community (Barra Caracciolo et al., 2020). Secondly, the olive tree presence not only increased the overall nematode population numbers, but also shifted community dominance from fungivores to bacterivores, a transition absent in bare soil. While fungal feeders (*Ditylenchus*) were once again the dominant group at high Ni contamination rates, the pattern changed at medium and low Ni concentrations, as bacterivorous nematodes surpassed fungivores in abundance, and were the dominant trophic group. The “rhizosphere effect” in the olive orchards likely explains these changes in the nematode community structure by mitigating the negative impacts of Ni contamination and by increasing the availability of microbial food resources that directly benefit the bacterivorous nematodes (Barra Caracciolo et al., 2020). This variability suggests that stochastic processes, such as microhabitat heterogeneity due to localized effects through soil nutrient enrichment from root exudates (Solá et al., 2019), significantly influenced the nematode community dynamics in the rhizosphere of olive trees. Despite facing increased heavy metal concentrations, olive trees exhibited resilience and contributed to improved soil health, fostering nematode abundance, particularly as Ni concentration decreased.

### Stochastic effects and variability in nematode responses

The predominance of stochastic forces is even more apparent in the differences between the responses of *Panagrolaimus* and *Acrobeles* at 40 and 70 ppm Ni, respectively. In olive orchard samples at 40 ppm, *Panagrolaimus* over-dominated, reflecting an opportunistic behavior driven by improved nutrient availability conditions rather than homogenizing responses to contamination (Kekelis et al., 2022). However, even though *Panagrolaimus* thrived under low Ni levels, it declined under higher contamination rates. This decrease of *Panagrolaimus* abundance at 70 ppm Ni demonstrates the genus’s susceptibility at moderate to elevated contamination levels, compared to the more tolerant bacterivorous genus *Acrobeles*. Martinez et al., (2019) suggested that different bacterivorous species have different pollutant tolerances, which influence their ecological interactions, then lead to a higher population fitness of one species under intensive pollution. With *Acrobeles* (cp-2: basal soil mesofauna) persisting in a wider range of Ni contamination rates and *Panagrolaimus* (cp-1: enrichment opportunist) prospering in less contaminated but nutrient-rich environments, these findings highlight the contrasting behaviors that nematodes of different cp scales represent. Moreover, these results show how cp scale-driven ecological roles affect nematode responses to contamination (Sánchez-Moreno & Navas, 2007).

The NMDS analysis and Simper test underline the distinct impacts of Ni contamination levels and olive tree presence on nematode community structure. The samples of bare soil at 70 ppm Ni, as well as those from olive orchards at 40 ppm Ni, were ordinated separately to all others. In the first case, the dominance of the fungivorous genus, *Ditylenchus*, is an indication of a simplified and stressed ecosystem; in the latter, the dominance of *Panagrolaimus*, reflected an enriched but less mature soil ecosystem. The clustering of communities in olive orchard samples at 70 ppm and 120 ppm Ni, as well as in those of bare soil at 40 ppm Ni, suggests that they follow a similar pattern. These communities were more similar and they exhibited higher alpha values (Renyi index), indicative of greater evenness in these systems. In these specific olive orchard soil samples, even though nutrient availability is high (Georgieva et al., 2002), the stress from Ni contamination plays a significant role in regulating the system, limiting the proliferation of opportunistic genera (Chauvin et al., 2020). In contrast, in the bare soil at 40 ppm Ni, the reduction of Ni stress allows more genera to increase in numbers, despite the limited availability of nutrients (Renčo et al., 2022).

These results align well with those related to the nematode community indices. The MI (maturity index) and EI (enrichment index) results demonstrated that nickel impact on soil food web dynamics is different depending on olive tree presence and contamination levels. Lower MI values in bare soil with high Ni levels indicated that the absence of plants intensifies the stress caused by pollution on the soil food web (Barra Caracciolo & Terenzi, 2021). *On the contrary, the presence of olive trees appeared to mitigate the adverse effects of increased nickel concentrations (*Zeng et al., 2018*), as reflected in higher values of the channel index (CI) and structure index (SI). It is important to note that higher CI values do not indicate better or worse condition; rather, they simply reflect a shift toward a fungal-dominated pathway of organic matter decomposition in the soil. In the absence of plants, the metabolic pathway tended to shift more strongly to fungal dominance.*

These results support the theory that vegetation, in our case olive trees, can mitigate the effects of heavy metal pollution by promoting a nutrient-rich environment that supports the diversity and survival of nematodes (Šalamún et al., 2017). Positioning olive trees as key agents in soil health restoration, this study emphasizes their role not only in nutrient cycling but also in fostering resilience within nematode communities under stress conditions. In uncontaminated Greek agroecosystems, soil nematode communities exhibit notably higher abundance and diversity, with total nematode numbers ranging from 901 to 1181 individuals per 100 ml of soil and genus richness spanning 34 to 58 genera (Tsiafouli et al., 2002). In contrast, Greek serpentine soils, which serve as a natural analogue for metal-stressed environments, support much lower nematode populations, with only 31–57 individuals per 100 ml and genus richness of 22–28 (Monokrousos et al., 2014). In our study, the presence of olive trees in Ni-contaminated soils substantially mitigated these negative effects: although total nematode populations and genus richness values were lower than those found in uncontaminated agricultural systems, however, they were higher than those observed in naturally contaminated (serpentine) soils. The importance of olive trees in reestablishing soil health in contaminated areas is shown by the increased nematode population and changes in community structure seen in olive orchards as opposed to bare soils.

### Interplay of deterministic and stochastic processes

Both stochastic and deterministic factors had a significant impact on nematode community dynamics and composition. Under high-stress conditions, deterministic parameters, such as Ni concentration and olive tree presence, exhibited comparatively predictable effects on nematode community diversity and abundance. On the other hand, the community is formed under more favorable conditions when stochastic elements are incorporated through diversity in genera-specific resilience and opportunistic behaviors. This implies a complex balance in which stochastic causes introduce variability within the framework of community structure, while deterministic processes establish the boundaries (Stamou & Papatheodorou, 2023). According to this interaction, stochastic components become more important under more favorable conditions, but deterministic processes may predominate under extreme stress. To deeper insights into community dynamics, future research should explore how these processes interact at smaller scales, including the microhabitat level.

## Conclusions

Our work has pointed out the sensitivity of soil nematode communities to Ni contamination and the mitigating role of vegetation, particularly olive trees. Observed patterns stress the importance of integrating both deterministic and stochastic frameworks in assessing the response of soil ecosystems to heavy metal stress. This dual approach provides a comprehensive perspective on ecosystem resilience, combining predictable trends with community variability under contamination. The results are of great value in developing workable strategies for sustainable management of soils, underpinning the potential of some plant species to improve the resilience and biodiversity of soils at contaminated sites. Practical applications could include the strategic use of specific vegetation types to promote nutrient cycling and support beneficial soil fauna in polluted areas. Further studies are recommended on the long-term relationships between plant-mediated effects, microbial community, and nematode dynamics for the optimization of remediation strategies in polluted soils. These future investigations should focus on the mechanisms driving plant-nematode-microbe interactions and their scalability for large-scale soil restoration projects.

## Data Availability

Data are available from the authors upon request.
